# Temperature Uniformity Control of 12-Inch Semiconductor Wafer Chuck Using Double-Wall TPMS in Additive Manufacturing

**DOI:** 10.3390/ma18010211

**Published:** 2025-01-06

**Authors:** Sohyun Park, Jaewook Lee, Seungyeop Lee, Jihyun Sung, Hyungug Jung, Ho Lee, Kunwoo Kim

**Affiliations:** 1 Daegyung Technology Application Division, Korea Institute of Industrial Technology, Daegu-si 42994, Republic of Korea; hyunypark@kitech.re.kr (S.P.); sylee94@kitech.re.kr (S.L.); jsung@kitech.re.kr (J.S.); 2 Department of Smart Mobility Engineering, Kyungpook National University, Daegu-si 41566, Republic of Korea; jaewk95@knu.ac.kr; 3 STACO Co., Ltd., Siheung-si 15084, Republic of Korea; hgjung@staco.kr; 4 Department of Robot and Smart System Engineering, Kyungpook National University, Daegu-si 41566, Republic of Korea

**Keywords:** wafer probe chuck, temperature uniformity, additive manufacturing, double-wall TPMS, thermo-fluid CFD analysis

## Abstract

In semiconductor inspection equipment, a chuck used to hold a wafer is equipped with a cooling or heating system for temperature uniformity across the surface of the wafer. Surface temperature uniformity is important for increasing semiconductor inspection speed. Triply periodic minimal surfaces (TPMSs) are proposed to enhance temperature uniformity. TPMSs are a topic of increasing research in the field of additive manufacturing and are a type of metamaterial inspired by nature. TPMSs are periodic surfaces with no intersections. Their continuous curve offers self-support during the additive manufacturing process. This structure enables the division of a single space into two domains. As a result, the heat transfer area per unit volume is larger than that of general lattice structures, leading to a superior heat transfer performance. This paper proposes a new structure called a double-wall TPMS. The process of creating a double-walled TPMS by adjusting the thickness of the sheet TPMS was investigated, and its thermal performance was studied. Finally, a double-wall TPMS was applied to the chuck. The optimal designs for the diamond and gyroid structures exhibited a difference in surface temperature uniformity of 0.23 °C and 0.66 °C, respectively. Accordingly, the models optimized with the double-wall TPMS are proposed.

## 1. Introduction

### 1.1. Semiconductor Wafer Chuck

Semiconductor integrated circuit devices are generally tested for their electrical properties at wafer level to determine whether the semiconductor chips are defective. During the wafer inspection process, the chuck holds the wafer using various methods such as electrostatic force or vacuum force. Subsequently, the electrical characteristics are tested at high, ambient, and low temperatures using a wafer probe. A passage for the cooling system is provided inside the chuck to allow the coolant to flow through and cool the wafer. Maintaining a uniform surface temperature is crucial because the overall surface temperature uniformity of the wafer affects the semiconductor inspection speed [1,2,3].

**Figure 1:**
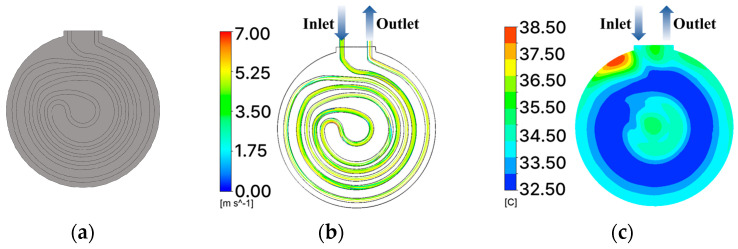
Traditional chuck with built-in cooling system: (**a**) serial type; (**b**) streamline; (**c**) contour of temperature.

Cooling systems in conventional chucks are divided into serial and parallel types based on the channel configuration. Figure 1a shows a conventional chuck with a cooling system in which the channel is configured as a serial type. In this type, the cooling channel forms a spiral from the outer edge of the chuck toward the center and then exits back at the outer edge. The channel is extended to increase the surface area. However, this increases the pressure difference between the inlet and outlet. Figure 1b,c show the streamlines of the velocity and temperature contours of the series type, respectively. The computational fluid dynamics (CFD) analysis was conducted at 30 LPM and 3000 W, resulting in a pressure loss of 0.8 bar and temperature difference of approximately 5 °C.

**Figure 2:**
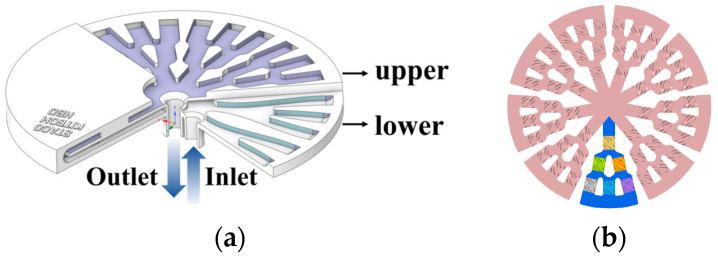
Radial structure chuck with built-in cooling system: (**a**) schematic of the 12-inch chuck showing the division into lower and upper sections; (**b**) radial structure of the cooling channels divided into nine parts for balanced coolant distribution.

To improve the temperature uniformity in conventional lower chucks, this study designed the cooling channels of a 12-inch chuck by dividing them into lower and upper sections, as shown in Figure 2a. The coolant flowed from the lower section to the upper section and spread into a nine-part radial structure, as shown in Figure 2b. The aim was to achieve a balanced flow distribution to improve the surface temperature consistency.

### 1.2. Introduction to Triply Periodic Minimal Surfaces

Inspired by the unique forms found in natural biological systems, cellular materials have been designed for various engineering applications. Cellular materials exhibit a range of functional characteristics, including morphological features, size, strength, weight, and thermal dispersion. They are shaped as truss structures, honeycomb structures, or porous materials, and have gained significant attention in various industries such as in aerospace, automotive, and medical applications [4,5,6,7,8,9]. However, the complexity of the shapes and defects that occur during the manufacturing process lead to difficulties in their application. Overcoming these challenges has led to the development of highly engineered metamaterials, and advancements in additive manufacturing (AM) have spurred increased research on these materials. They exhibit unique geometric, physical, and mechanical properties. Recently, metamaterials based on triply periodic minimal surfaces (TPMSs) have been increasingly recognized [10,11,12,13,14,15,16,17,18,19].

**Figure 3:**
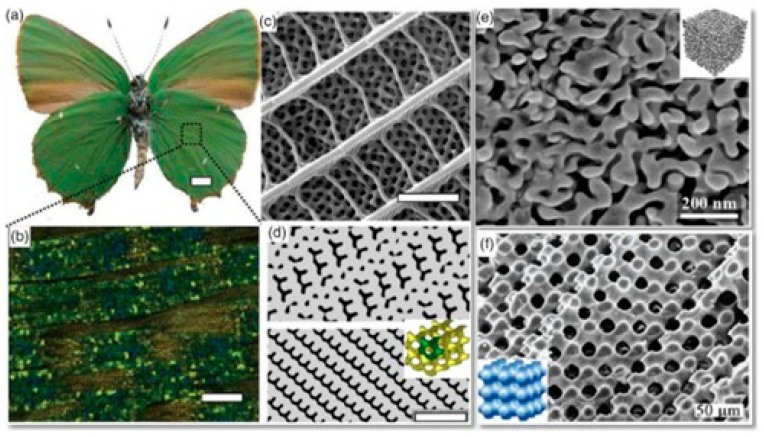
Examples of TPMSs found in nature [20].

**Figure 4:**
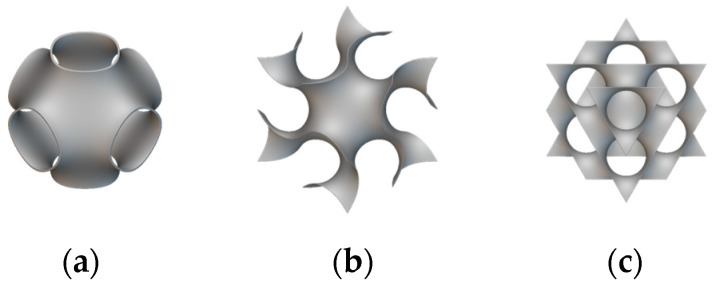
Types of TPMS: (**a**) primitive; (**b**) gyroid; (**c**) diamond.

As shown in Figure 3 [20], a TPMS is a structure observed in natural examples, such as soap films, block copolymers, butterfly wing scales, and sea urchins [21]. TPMSs include various types of structures, with the most common being primitive, gyroid, and diamond, as shown in Figure 4. These structures locally minimize the surface area, resulting in a surface where the mean curvature is zero at every point. This property implies that the sum of the principal curvatures at each point is zero, meaning that these surfaces are equally convex and concave, forming saddle-like or hyperbolic shapes. They are termed ‘minimal’ because, given a fixed boundary curve, the area of a minimal surface is extremal compared to other surfaces. Furthermore, these surfaces exhibit a crystalline structure that repeats periodically in three dimensions, known as triply periodicity [22]. TPMSs are also self-supporting structures, enabling additive manufacturing without the need for additional supports. Due to their unique geometric characteristics, TPMS structures are easily produced through additive manufacturing techniques, as they are challenging to produce using conventional manufacturing methods. These surfaces are smooth with no sharp edges, and the two domains created by the curved structure are independently organized without intersections [23]. The separated domains have the unique characteristic of being repeated periodically and infinitely in three directions. The surfaces of a TPMS are represented using implicit functions. Implicit surfaces are defined as iso-surfaces of a function, where the sign of the function distinguishes points inside or outside the surface, while the equality specifies the thickness of the implicit surface. Equations (1)–(3) describe the surfaces of the primitive, gyroid, and diamond structures in the form of implicit functions, generated through combinations of trigonometric functions.

(1)
Primitive
      cos⁡(x)+cos⁡(y)+cos⁡(z)=ϕ

(2)
Gyroid
  sin⁡(x)cos⁡(y)+sin⁡(y)cos⁡(z)+sin⁡(z)cos⁡(x)=ϕ

(3)
Diamond
  cos⁡(x)cos⁡(y)cos⁡(z)−sin⁡(x)sin⁡(y)sin⁡(z)=ϕ

**Figure 5:**
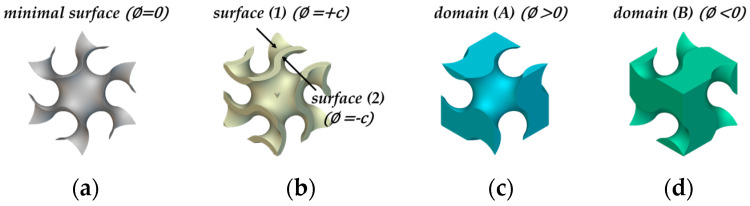
Unit cell of gyroid topologies: (**a**) minimal surface; (**b**) sheet-TPMS; (**c**) and (**d**) solid-TPMS.

A constant ϕ is used to create a surface with an offset value. At ϕ=0, as shown in Figure 5a, the minimal surface generates two subdomains with equal volumes that separate the given space. Constant *c* is the offset value. Figure 5b shows the sheet TPMS generated with *c.* The sheet thickness was generated by offsetting the two surfaces of the ϕ=±c normal to the ϕ=0 surface in two opposite directions. A sheet TPMS of thickness is created by filling a thin channel between two offset surfaces [24,25]. In contrast, if the regions separated by the minimal surface are used as domains (A) and (B), the TPMS is referred to as a solid TPMS (Figure 5c,d).

Thus, TPMS-based structures can be used in applications such as catalytic reactors, fuel cells, batteries, and heat exchangers, owing to their large surface areas [26,27]. This study presents a method for enhancing the temperature uniformity of semiconductor wafer chucks using sheet TPMS structures. Section 2 provides an introduction and theoretical background to the new structure, known as the double-wall sheet TPMS. Section 3 proposes an optimal design model for a semiconductor chuck with superior temperature uniformity using a double-wall sheet TPMS. 

## 2. Double-Wall Sheet TPMS

### 2.1. Concept of Double-Wall Sheet TPMS

**Figure 6:**
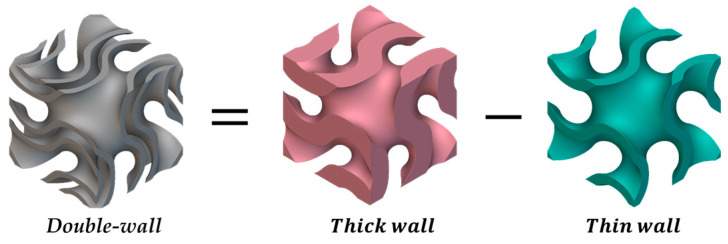
Design process of the proposed double-wall sheet TPMS.

This study proposes a new structure termed a double-wall sheet TPMS to enhance the characteristics of an increased surface area, facilitating heat transfer per unit volume. The double-wall sheet TPMS is a structure created by removing the TPMS with a smaller *c* value from the TPMS with a larger value of *c.* The value of *c* represents the geometric characteristics of the structure, which alter the configuration between the channels. Figure 6 shows an overview of the design process for the double-walled sheet TPMS, and Equations (4) and (5) show the process mathematically. The TPMS modeling was performed using nTop software.

(4) $$\begin{array}{cc} \mathrm{Single}-\mathrm{wall}\;\mathrm{Sheet}\;\mathrm{TPMS} & \;f(x,y,z)=c_{thick}\;g(x,y,z)=c_{thin} \end{array}$$

(5) $$\mathrm{Double}-\mathrm{wall}\;\mathrm{Sheet}\;\mathrm{TPMS}\;h(x,y,z)=f(x,y,z;c_{thick})-g(x,y,z;c_{thin})$$

**Figure 7:**
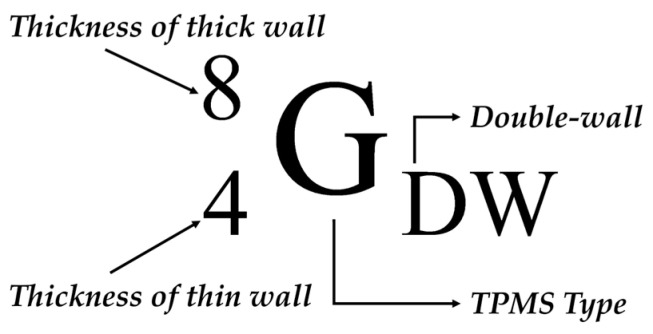
Notation for single- and double-walled TPMS.

Figure 7 shows the notation for the single and double structures. An uppercase letter represents the TPMS type: G for gyroid and D for a diamond structure. The subscript DW on the right represents the double wall, and SW denotes a single wall. The upper-left superscript represents the thickness of the sheet TPMS $c_{\mathrm{thick}}$, whereas the lower subscript $c_{\mathrm{thin}}$ denotes the thickness of the removed sheet TPMS.

Table 1 lists the changes in the volume fraction and surface area of the single- and double-wall structures of the gyroid and diamond. In the single structure, as *c* increases, the surface area tends to decrease. In contrast, in the double structure, as the value of $c_{\mathrm{thin}}$ increases, both the volume fraction and the surface area tend to increase.

Table 2 and Table 3 compare the flow cross-sectional areas of the single and double structures of the gyroid and diamond, showing the shape changes in the flow cross-section according to the flow direction along the x-axis. The blue and white regions represent fluid and solid regions, respectively. It can be observed that the expansion of the number of domains in the double structure of both the gyroid and diamond resulted in the creation of an additional fluid area when compared to the single structure. This is a factor that affects the fluid velocity and pressure loss.

The double-wall sheet TPMS generated through this process expands the three domains, in contrast to the two domains in the existing single-wall sheet TPMS, resulting in an increase in both the volume fraction, which represents the space through which fluid can be low per unit volume, and the surface area for the transfer occurring per unit volume. Using these characteristics, a double-wall TPMS was applied to a model with a constant heat flux input to enhance heat transfer performance using a cooling channel system.

### 2.2. Thermo-Fluid Trends of Double-Wall Sheet TPMS

#### 2.2.1. Thermo-Fluid CFD Analysis

To evaluate the heat transfer performance of the single- and double-wall structures, a thermal fluid CFD analysis was conducted using Fluent software. The mesh required for the simulation was generated using HyperWorks software. The analytical conditions are listed in Table 4. Prior to the CFD simulation, the Reynolds number was calculated to examine the fluid characteristics. Generally, when the Reynolds number of the flow inside the pipe is less than 2300, a laminar flow is considered; when it is between 2300 and 4000, a transitional flow is considered; and when it is more than 4000, a turbulent flow is considered. The hydraulic diameter $D_{h}$ required to calculate the Reynolds number is defined as

(6) $$D_{h}=\frac{4A_{c}}{P},$$

where $A_{c}$ is the cross-sectional area through which the fluid flows and *P* (wetted perimeter) refers to the length of the boundary in contact with the fluid when viewed from the cross-section. However, because a TPMS was used in this study, the fluid section in the x-axis direction was not constant and changed. Therefore, the hydraulic diameter $D_{h}$ for calculating the Reynolds number related to the TPMS was computed as follows [28,29]:

(7) $$D_{h}=\frac{4V}{A_{s}},$$

where *V* is the volume of the fluid and $A_{s}$ is the surface area where the TPMS and fluid interact. Finally, the Reynolds number for the TPMS structure is defined as

(8) Re⁡=ρυDhμ,

where *ρ* is the density of the fluid, *υ* is the velocity of the fluid, and *μ* is the viscosity. The shear stress transport (SST) k-omega model was used for turbulence, and the Transition SST model was used for the analysis of the transition region.

After analyzing the Reynolds number and selecting an appropriate analysis model, CFD was performed. Based on the CFD results, the Nusselt number was used as a performance indicator to compare the heat transfer performances of the single and double structures. The Nusselt number is a dimensionless number that represents the relative ratio of the conductive heat transfer within a fluid to the convective heat transfer occurring at the surface of the geometry. Therefore, the thermal conductivity of the fluid, $k_{f}$, is used. The average Nusselt number is typically calculated as follows:

(9) $$\bar{Nu}=\frac{\bar{h}D_{h}}{k_{f}}$$

In complex geometries, such as TPMS, the average convection coefficient $\bar{h}$ varies depending on the location, and the local convection coefficient h is different at each location. To determine $\bar{h}$, it is necessary to integrate h over the surface, as shown in Equation (10). The local convection coefficient h was calculated using Equation (11) related to local surface heat flux $q^{\prime \prime }$ based on Newton’s law of cooling.

(10) $$\bar{h}=\frac{1}{A_{s}}\int_{A_{s}}hdA_{s}$$

(11) $$q^{\prime \prime }=h(T_{s}-T_{\infty })$$

In this study, h was calculated at each coordinate using the extreme values of $T_{wall}(x,y,z)$ and $q^{\prime \prime }(x,y,z)$ from the CFD analysis results, as shown in Equation (12). In this case, $T_{\infty }$ is 300 K.

(12) $$h(x,y,z)=\frac{q^{\prime \prime }(x,y,z)}{T_{wall}(x,y,z)-T_{\infty }}$$

**Figure 8:**
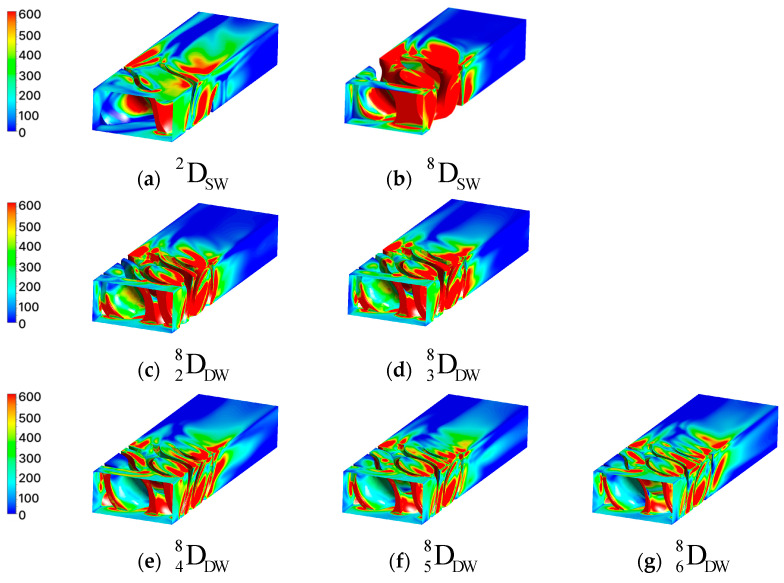
Contour of Nusselt number in the diamond structures.

After summing all h, it was divided by the total number of coordinates to obtain $\bar{h}$ (Equation (12)). It can be used more easily than integrating the complex geometry of the TPMS. The average Nusselt number was calculated by substituting $\bar{h}$ into Equation (9).

(13) $$\bar{h}=\frac{\sum_{1}^{N}h(x,y,z)}{N}$$

where *N* is the total number of coordinates. Finally, to visually confirm the convective heat transfer performance according to the TPMS geometry, the local Nusselt number at each coordinate was calculated using Equation (14) and visualized, as shown in Figure 8. In the Nusselt number contour, the red regions indicate areas with high Nusselt numbers, signifying that convective heat transfer dominates over conductive heat transfer within the fluid. Conversely, the blue regions represent areas where conduction is the dominant mode of heat transfer.

(14) $$Nu(x,y,z)=\frac{h(x,y,z)D_{h}}{k_{f}}$$

#### 2.2.2. CFD Results of Diamond Structure

**Figure 9:**
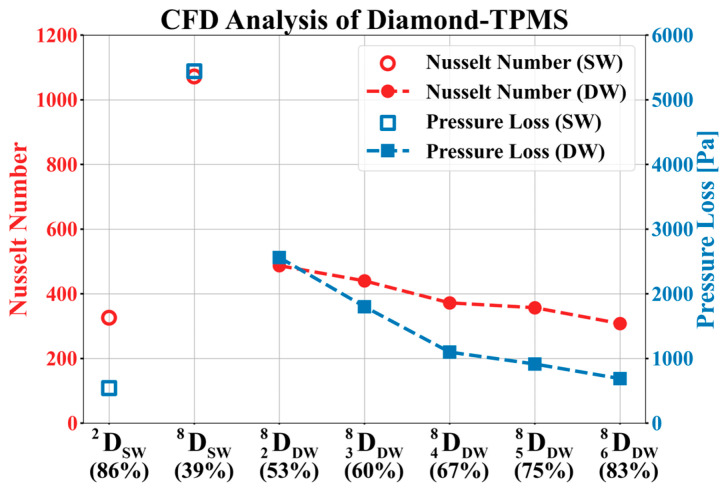
Variation in Nusselt number and pressure loss with respect to the volume fraction of diamond.

Table 5 lists the CFD results and fluid characteristics of the diamond structure. Figure 9 shows the relationship between the Nusselt number and pressure loss in a diamond consisting of single and double structures. The Reynolds number for each sample is also presented, and the percentage values on the x-axis represent the volume per unit of each structure.

In the single-wall diamond, the Nusselt number increased rapidly from 326 to 1072. This makes it difficult to precisely tune the Nusselt number for specific heat transfer reactions. The pressure loss also increased rapidly from 544 to 5444 Pa, which limited its control of the pressure loss. In other words, it is difficult to optimize the pressure loss of a single structure while improving its heat transfer performance.

In the double-wall diamond, the Nusselt number gradually decreased as the volume fraction increased. The pressure loss also gradually decreased, similar to the Nusselt number. This implies that the heat transfer performance can be controlled by a low pressure drop. The Nusselt and pressure losses of the double-wall diamond area were located between those of the single wall, providing adequate heat transfer performance while maintaining a lower pressure loss than that of the single wall. The double-wall diamond has advantages for system optimization, as it provides design flexibility to balance the heat transfer performance and pressure loss.

The change in the Nusselt number was analyzed using two factors. The first reason for this was the change in the vertical impact area. When the fluid collides vertically with the structural surface, the boundary layer is regenerated, which reduces the heat transfer resistance and generates turbulence, resulting in a thinner boundary layer. This enhances the convective heat transfer performance at the surface of the structure. For the double-wall diamond, as the volume fraction decreased, the vertical collision area also decreased; therefore, the Nusselt number tended to decrease. The single structure DSW8 exhibited the highest Nusselt number because it had the largest vertical impact area.

The second reason is the change in the cross-sectional area of the fluid. In the previous section, it was confirmed that as the volume fraction of the TPMS increased, the fluid cross-sectional area also increased. According to the continuity equation, as the fluid cross-section increases, the fluid velocity decreases. A decrease in the velocity leads to a reduction in the inertial forces and an increase in the boundary layer thickness. Consequently, the heat transfer effect due to convection was reduced. Conversely, as the sectional area decreases, the velocity increases, and the effect of the inertial force increases, improving the heat transfer performance compared with conduction. In addition, if the velocity changes due to changes in the sectional area, it affects the Reynolds number, which represents the ratio of inertial forces to viscous forces. Therefore, as the volume fraction of the double structure increases, the Reynolds number decreases.

#### 2.2.3. CFD Results of Gyroid Structure

**Figure 10:**
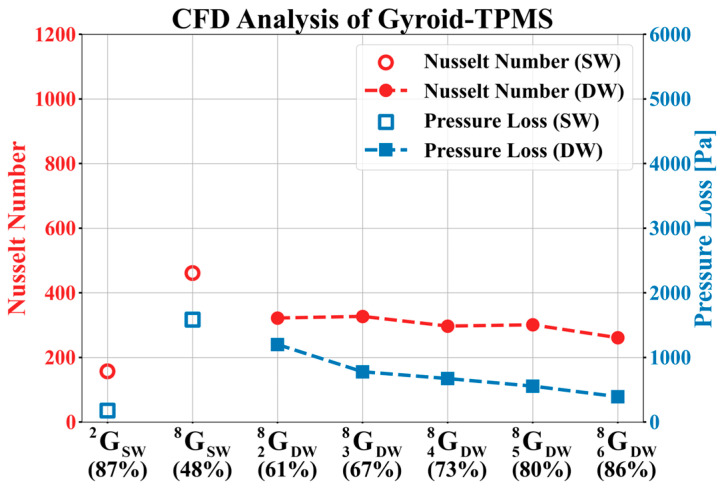
Variation in Nusselt number and pressure loss with respect to the volume fraction of gyroid.

Table 6 lists the CFD results for the gyroid structure. Figure 10 shows the trends observed in the CFD results. In the gyroid, similar to the diamond structure, the pressure loss, Reynolds number, and Nusselt number of the double structure tended to decrease as the volume fraction increased. However, in GDW38 and GDW58, the Nusselt number increased by 5 and 4, respectively, compared to the previous model. The increase in the Nusselt number is attributed to the changes in the fluid region owing to the coordinate shift during the modeling process of the basic specimen for the gyroid. A mismatch can occur between the modeled shape and mesh in complex geometries, such as gyroids, during the mesh generation process after modeling. To avoid this mismatch, the geometry must be modified using coordinate shifts.

**Figure 11:**
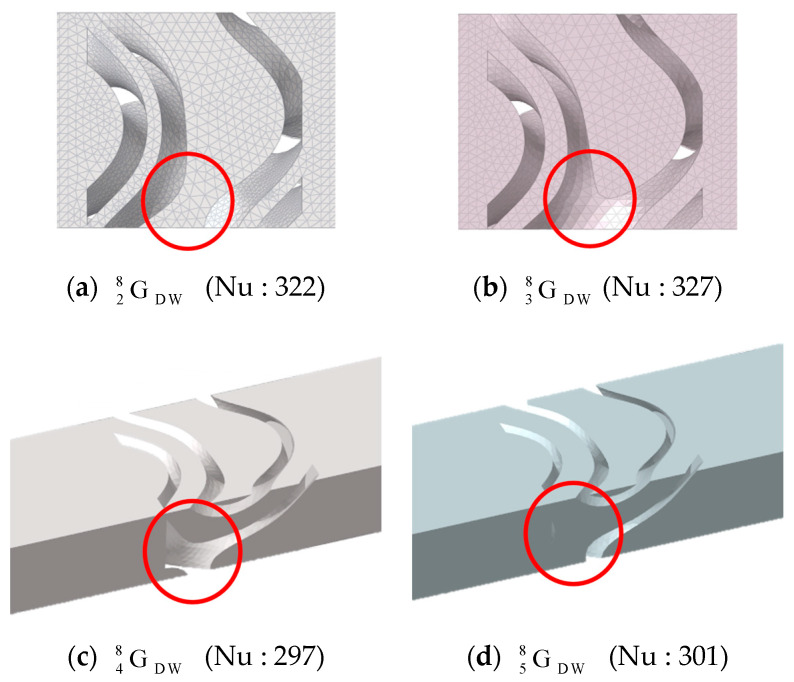
Shape difference due to coordinate shifts in the gyroid.

**Figure 12:**
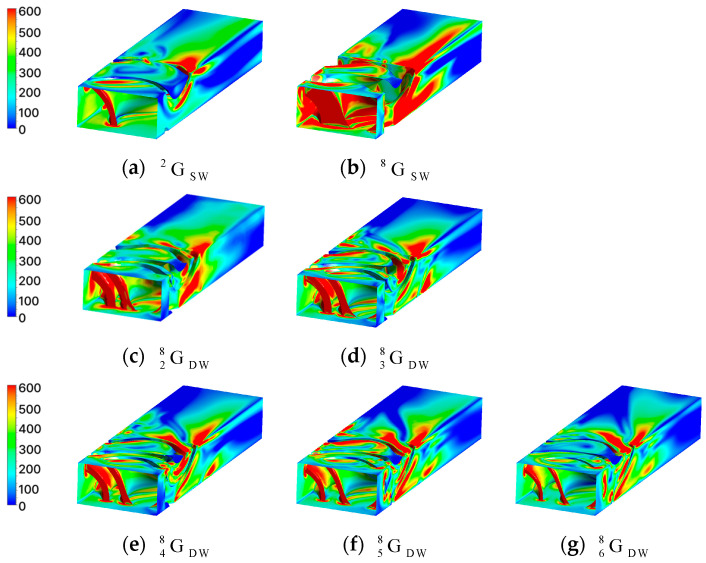
Contour of Nusselt number in the gyroid structures.

The red circular area in Figure 11a GDW28 shows the dead zone, where the fluid does not flow. The dead zone contributed to a decrease in the Nusselt number. However, in GDW38 (Figure 11b), the absence of the dead zone meant it was not included in the Nusselt number calculation. As a result, the Nusselt number increased by five compared to GDW28, even though the volume fraction of GDW38 increased. This difference can be visually confirmed from the contours of the Nusselt number shown in Figure 12c,d.

Comparing the red areas in GDW48 (Figure 11c) and GDW58 (Figure 11d), it is observed that a region where the fluid flows very narrowly is formed in GDW58 leading to an increase in fluid velocity. The convection effect was enhanced due to the increase in the velocity, which can be also visually confirmed through the Nusselt contours in GDW48 (Figure 12e) and GDW58 (Figure 12f). As a result, the Nusselt number increased by four despite the increase in volume fraction.

This study addressed the mesh mismatch that occurs during the mesh generation process after modeling a gyroid by modifying the geometry through coordinate shifts. Consequently, an increase in the Nusselt number was observed. However, this was a consequence of the unavoidable modifications made to the analysis. It was anticipated that under the same modeling conditions without geometric modifications, the Nusselt number would exhibit a decreasing trend.

## 3. Optimal Design of the 12-Inch Chuck

### 3.1. Radial Structure of the 12-Inch Chuck 1/9 Model

**Figure 13:**
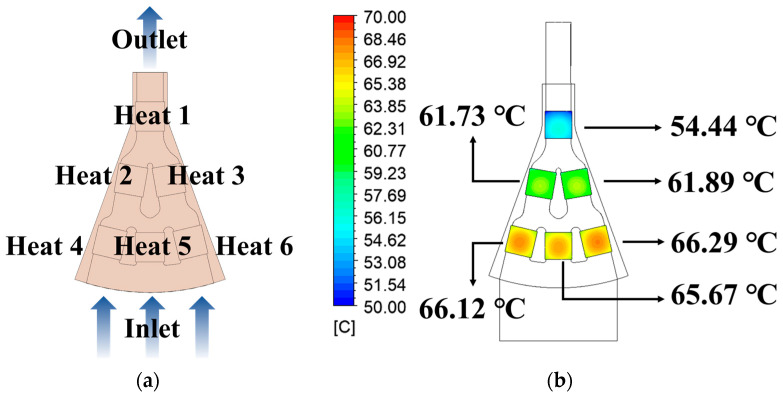
The 1/9 model of the 12-inch chuck: (**a**) geometry of 1/9 model without TPMS; (**b**) CFD results.

In this study, the channel of a 12-inch lower chuck was structured radially, divided into nine branches, and exhibited axial symmetry. Therefore, the analysis was conducted on the 1/9 model shown in Figure 13a. The 1/9 model was equipped with six thermoelectric modules (TEMs) arranged in configurations of three, two, and one according to the flow direction from the inlet to the outlet. The regions where the TEMs are located are designated as Heat 1 to Heat 6.

To verify the thermo-fluid trends of the 1/9 model, CFD analysis was conducted without the TPMS. Table 7 lists the analysis conditions for the entire 12-inch chuck. The analysis results for the 1/9 model, calculated under 12-inch conditions, are the same as those in Table 4 (Section 2). Figure 13b shows the CFD results. The surface average temperature at Heat 1 was the lowest at 54.44 °C, whereas Heat 6 exhibited the highest temperature at 66.29 °C. The temperature difference between Heat 1 and Heat 6 was confirmed to be 11.85 °C.

In Heat 1, the total flow rate flowed, whereas in Heats 4–6, the flow rate was three times smaller, resulting in a difference in average temperature. Heats 2 and 3 were located in the intermediate region, and both the flow rate and average temperature were calculated to have values between those of Heats 1 and 4 to 6. These differences in the flow rates act as factors influencing the heat transfer performance in each region.

In regions with low mass flow rates, such as between Heats 4 and 6, it is essential to select a TPMS model with excellent heat transfer performance. Conversely, in regions with high mass flow rates, such as Heat 1, it is suitable to select a model with a lower heat transfer performance. This is because using a model with a high heat transfer performance in Heat 1 can result in unnecessary overcooling. To enhance the temperature uniformity across the heated regions, it is essential to conduct an optimal design that considers the mass flow rates.

### 3.2. Optimal Design Using Double-Wall Sheet-Diamond

#### 3.2.1. Selection of Design Variables for Heat 1

For Heat 1, because the largest amount of flow occurred, Type (1) DSW1 and Type (2) DSW2 were selected from among the thin single-wall sheet TPMS with lower heat transfer performance to maintain temperature uniformity. For Heats 2–6, different structures were arbitrarily assigned, and the CFD analysis was conducted when DSW1 and DSW2 were placed in Heat 1. This approach aims to preselect a model with high temperature uniformity to ensure optimal heat transfer performance.

Table 8 presents the CFD analysis results obtained using DSW1 and DSW2. The average temperature difference in the region where the TEMs are placed in DSW1 was 0.86 °C, which is lower than the 1.56 °C observed in DSW2. Placing DSW1 in Heat 1 indicated that the temperature distribution within the system became more uniform. To prevent local overcooling owing to the increased flow rate, it is appropriate to select DSW1 for Heat 1.

#### 3.2.2. Discrete Latin Hypercube Design

In the cases of Heats 4–6, an approximately three-times-lower flow rate is observed compared to Heat 1. Considering this, the model with the highest heat transfer performance of the double structure presented in Section 2 was used as a design variable. It was confirmed that the models DDW28 and DDW38 have high Nusselt numbers. For Heats 2 and 3, models DDW58 and DDW68 with lower Nusselt numbers were designated as the design variables.

An optimal design using a general experimental design method requires the analysis of all combinations of variables using a full-factorial approach. Table 9 lists the design variables for Heats 1–6. Each heat region had two design variables and $2^{6}$ analyses were required. This approach is inefficient because it requires a significant amount of time for simulation and experiments.

DSW1, DSW2GDW58, GDW68GDW58, GDW68GDW28, GDW38GDW28, GDW38GDW28, GDW38 Latin Hypercube Sampling (LHC) is a technique developed to address the limitations of Monte Carlo sampling, which relies on random sampling from the design space. The Monte Carlo method often results in sample points being concentrated in specific regions of the design space and requires a large number of samples, leading to high computational costs. In contrast, LHC divides the design space into equal probability intervals and extracts sampling points based on the random global technique. LHC is effective for problems involving continuous design variables [30,31,32]. However, this study adopts the double-wall sheet TPMS structure, analyzed for thermo-fluid characteristics in Section 2.2, to construct the design table. Since the design variables in this case are discrete rather than continuous, the Discrete Latin Hypercube Sampling (DLHC) technique was applied [33].

Table 10 presents the equations for the optimal design. Table 11 shows the DLHC-based design of experiments (DOE) table constructed using the EasyDesign software (version 2023). However, since Heat 1 was set with DSW1 as a fixed design variable in Section 3.2.1, the DLHC technique was applied only to Heats 2–6. The design variables were the TPMS applied to each heating region. The objective function was defined to minimize the difference between the maximum and minimum average surface temperatures at the proposed design points. Temperature uniformity was ensured by minimizing the objective function, and an optimal design was achieved with minimal sampling using the DLHC technique.

#### 3.2.3. Sequential Approximate Optimization

Based on the results obtained through sampling data based on the DLHC, a surrogate model was constructed, and a sequential approximate optimization (SAO) process was performed to find the optimal solution. The SAO performs simulations at the sampling points extracted through the DLHC, and then reconstructs the surrogate model based on the data. The DLHC and SAO enabled us to continuously improve the design performance while minimizing the CFD analysis.

Table 12 presents the SAO results. In the second stage of SAO, the response function converged at 0.23 °C. An optimized model that maximized the target temperature uniformity was derived using SAO.

#### 3.2.4. Verification of Temperature Distribution Using Boxplot

**Figure 14:**
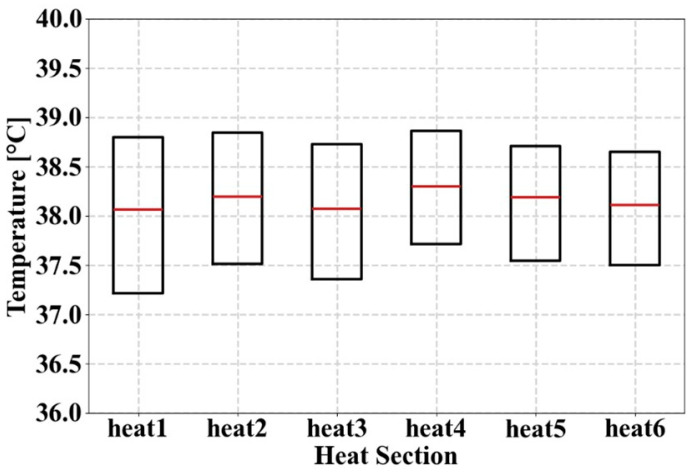
Boxplot for SAO 2 applied to the diamond structure.

**Figure 15:**
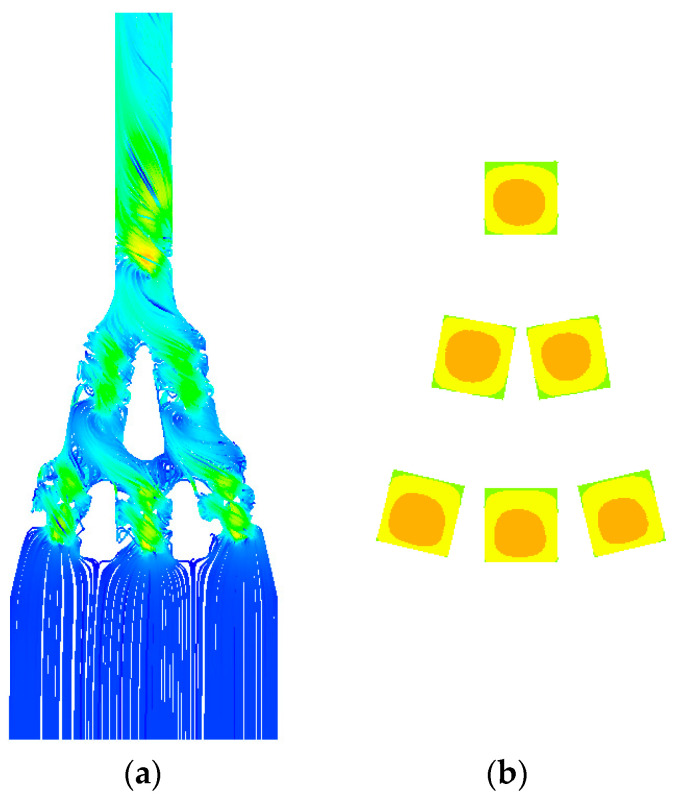
The 1/9 model optimally designed with double-wall diamond: (**a**) streamlines; (**b**) temperature contour.

To evaluate the temperature uniformity, the temperature data were analyzed using a boxplot. Table 13 shows the mean temperature, median, and interquartile range (IQR) to provide an intuitive view of the data distribution. Figure 14 shows a boxplot of the median and IQR. Figure 15 displays the flow of the optimally designed model and the temperature distribution for Heats 1 to 6.

The smaller the difference between the average and median temperatures, the more symmetrically the data are distributed. In the case of Heat 1, the average temperature was 38.16 °C, and the median was 38.07 °C, resulting in a difference of 0.09 °C. The differences for Heats 2 to 6 were also all below 0.2 °C, indicating that the temperature data were symmetrically distributed. Therefore, it was reasonable to use the average temperature of Heats 1–6 as a representative value and to converge such that their maximum and minimum values were minimized because the data were not biased.

The IQR indicates the extent to which the middle 50% of the data is spread out and is calculated as the difference between Q3 (third quartile) and Q1 (first quartile). A smaller IQR value indicates that 50% of the data are concentrated around the median. In the case of Heat 1, the middle 50% of the data is distributed within a range of 1.58 °C. For Heats 2 and 3, the temperature differences were distributed within approximately 1.35 °C, and in the case of Heats 4 to 6, the temperature was distributed within approximately 1.15 °C. An IQR value of 1.15 °C indicates very low variability in temperature and suggests that the data are concentrated around the median. In this study, an IQR value of 1.5 °C or less was judged as a criterion for excellent temperature uniformity.

Through boxplot analysis, the symmetry and concentration of the temperature data were evaluated, revealing that the difference between the average temperature and the median was less than 0.2 °C, and the IQR value was also below 1.5 °C, confirming that the temperature was evenly distributed. This confirms the excellent temperature uniformity and that the optimal design model in this study was a valid result for optimizing temperature uniformity. A boxplot analysis was used to evaluate the symmetry and concentration of the temperature data, confirming that the temperatures were uniformly distributed.

In conclusion, the SAO 2 model [DSW1, DDW68, DDW68, DDW28, DDW38, DDW28] is proposed as the optimal design (Table 12), which can maintain the pressure loss at 636 Pa with high temperature uniformity by converging the difference in the average temperature value between each heat area to 0.23 °C.

### 3.3. Optimal Design Using Double-Wall Sheet Gyroid

#### 3.3.1. Selection of Design Variables for Heat 1

The optimal design using the gyroid structure aims to preselect the design variables for Heat 1, where the highest flow rate occurs, similar to that of the diamond structure. To achieve this, a CFD analysis was conducted on GSW1 and GSW2, and the results are presented in Table 14. When using Type (1) GSW1, the difference between the maximum and minimum average temperatures across the regions was 0.78 °C. In contrast, for Type (2) GSW2, this difference was 1.98 °C, which is 1.2 °C higher than that of Type (1). Considering the temperature uniformity, GSW1 was selected for Heat 1.

#### 3.3.2. Discrete Latin Hypercube Design

Table 15 shows the DOE using the DLH by specifying the design variables for Heats 2 to 6. Considering the heat transfer performance according to the difference in flow rate at each location, similar to the diamond, Heats 4 to 6 were specified as design variables GDW28 and GDW38, which have high heat transfer performance, and Heats 2 and 3 were specified as the design variables GDW58 and GDW68, respectively.

#### 3.3.3. Sequential Approximate Optimization

The SAO process was performed to determine the optimal design model in which the response function converged to the minimum value using a surrogate model constructed based on the DLHC. In the SAO 2, it converged to 0.66 °C (Table 16), which was the same value as the response function of Point 6 of DLHC.

#### 3.3.4. Verification of Temperature Distribution Using Boxplot

**Figure 16:**
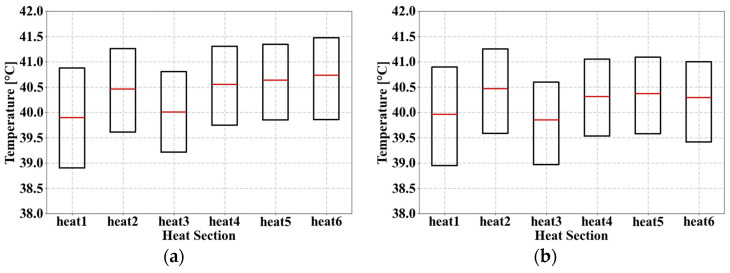
Boxplot applied to the gyroid: (**a**) Point 6 and (**b**) SAO 2.

Although the response function values of Point 6 and SAO 2 are both 0.66 °C, differences were observed in the design variable configurations for Heats 4 to 6. Therefore, a comparative boxplot analysis was conducted for the two models to determine the optimal design model aimed at improving temperature uniformity (Figure 16).

Table 17 shows the difference between the average and median temperatures and the IQR value of Point 6. In Point 6, the difference between the average temperature and the median varied from 0.10 °C to 0.33 °C, whereas in SAO 2 (Table 18), it varied from 0.10 °C to 0.27 °C. This indicates that the temperature data distribution at SAO 2 was more symmetrical than that at Point 6. In addition, the difference between the maximum and minimum medians was 0.83 °C in Point 6 and 0.61 °C in SAO 2. By comparing the temperature uniformity from Heats 1 to 6, it can be observed that SAO 2 shows a more even distribution than Point 6.

The IQR values were compared to evaluate the concentration of the median temperature data. The IQR values of Point 6 varied from 1.49 °C to 1.97 °C, and the IQR values of SAO 2 varied from 1.51 °C to 1.95 °C. In Heat 5, the IQR value for Point 6 was 0.02 °C lower than that of SAO 2, whereas it was 0.02 °C higher than that of SAO 2 for Heat 1. This comparison indicated that the temperature concentration at certain intervals for Point 6 was lower.

**Figure 17:**
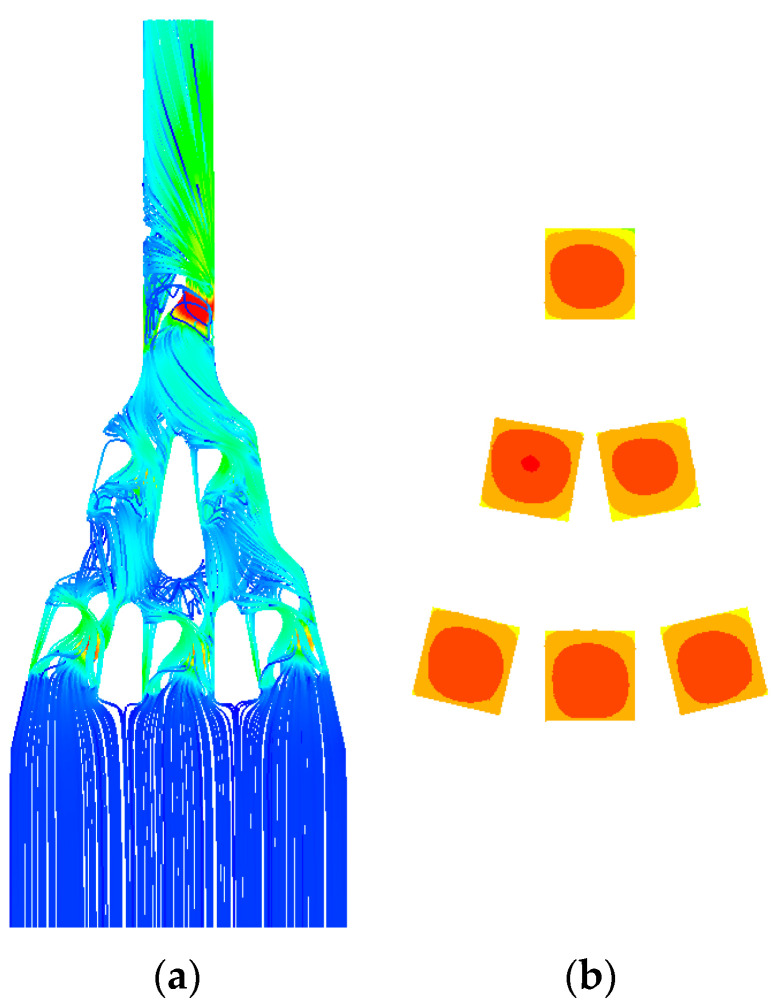
The 1/9 model optimally designed with double-wall gyroid: (**a**) streamlines; (**b**) temperature contour.

In conclusion, the SAO 2 model demonstrated a higher performance than that of Point 6 in terms of the symmetry of the temperature data distribution and concentration around the median. Therefore, the SAO 2 model [GSW1, GDW68, GDW68, GDW28, GDW28, GDW28], which utilizes the double-wall gyroid in the 1/9 model, is proposed as the optimal design for enhancing temperature uniformity (Figure 17).

## 4. Conclusions

This paper presents a new double-wall sheet TPMS based on TPMS structures inspired by nature. The effect of the structural characteristics of the double-wall sheet TPMS on the thermos-fluid performance was analyzed.

Compared with the single-wall sheet TPMS, the surface area per unit volume of the double-wall sheet TPMS increases, which means that the heat transfer surface area interacting with the fluid is expanded. In addition, as the volume fraction of the fluid occupying the unit volume increased, the pressure loss tended to decrease compared with that of the single-wall sheet TPMS. The thermo-fluid performance associated with these structural characteristics is as follows:

In the case of a single-wall sheet diamond, the Nusselt number increases rapidly as the thickness increases, whereas in the case of a double-wall sheet diamond, the Nusselt number increases gradually, even as the thickness increases (i.e., the volume fraction decreases). This means that the double-wall sheet diamond can more precisely adjust the heat transfer performance and has the advantage of maintaining a lower pressure loss than a single structure. Therefore, double-wall diamond structure is advantageous in terms of maintaining a balance between the heat transfer performance and pressure loss.

For the gyroid, the geometry was modified through coordinate translation to resolve the CFD analysis error caused by the mismatch between the model and the mesh. As a result, the Nusselt number tended to increase in some sections but was expected to show the same tendency as a diamond when a geometrically identical gyroid model was used.

Based on the thermo-fluid characteristics of the double-structured TPMS, an optimal design was developed to maintain uniform surface temperatures in the 1/9 model of the semiconductor lower chuck. Considering the differences in the heat transfer performance based on the mass flow rates, the appropriate diamond and gyroid models were designated as design variables for each heat region. Through the DLHC method and SAO process, the optimal design was derived with minimal sampling data, which served as the basis for the CFD analysis. The temperature data for each heat region were compared using a boxplot. The optimal design using the diamond resulted in a difference in surface temperature uniformity of 0.23 °C and a pressure loss of 636 Pa. The optimal design results using the gyroid were 0.66 °C and 517 Pa. The 1/9 model optimized with the double-wall TPMS is proposed as follows:

**Diamond:**

[DSW1, DDW68, DDW68, DDW28, DDW38, DDW28]

**Gyroid:**

[GSW1, GDW68, GDW68, GDW28, GDW28, GDW28]

## Figures and Tables

**Table 1:** Structural features of gyroid and diamond.

	Single	Surface [mm^2^]	Volume Fraction [%]		Surface [mm^2^]	Volume Fraction [%]
**1**	GSW2	2022	87	DSW2	2174	86
**2**	GSW8	1689	48	DSW8	1718	39
	**Double**	**Surface [mm^2^]**	**Volume Fraction [%]**		**Surface [mm^2^]**	**Volume Fraction [%]**
**1**	GDW28	2655	61	DDW28	2747	53
**2**	GDW38	2690	67	DDW38	2803	60
**3**	GDW48	2723	73	DDW48	2860	67
**4**	GDW58	2756	80	DDW58	2919	75
**5**	GDW68	2789	86	DDW68	2985	83

**Table 2:** Flow sectional area for the gyroid structure along the x-axis.

Position [mm]	x = 0	x = 3	x = 6	x = 9	x = 12
**Section Area** **(** GSW8 **)**	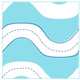	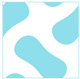	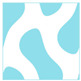	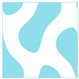	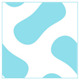
**Area [mm^2^]**	477	407	426	426	407
**Position [mm]**	**x = 0**	**x = 3**	**x = 6**	**x = 9**	**x = 12**
**Section Area** **(** GDW48 **)**	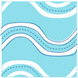	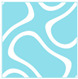	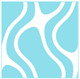	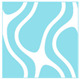	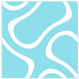
**Area [mm^2^]**	673	683	636	636	683

**Table 3:** Flow sectional area for the diamond structure along the x-axis.

Position [mm]	x = 0	x = 3	x = 6	x = 9	x = 12
**Section Area** **(** DSW8 **)**	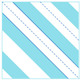	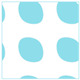	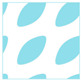	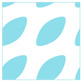	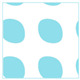
**Area [mm^2^]**	410	307	321	321	307
**Position [mm]**	**x = 0**	**x = 3**	**x = 6**	**x = 9**	**x = 12**
**Section Area** **(** DDW48 **)**	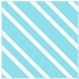	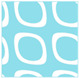	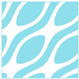	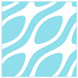	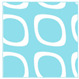
**Area [mm^2^]**	632	656	564	564	656

**Table 4:** Condition for thermal fluid analysis.

**Inlet mass flow**	3.33 LPM
**Inlet temperature**	26.9 °C
**Outlet Pressure**	0 bar
**Heat flux**	126,263 W/m^2^

**Table 5:** Flow features and CFD results of the diamond structure.

	Single	$D_{h}$ [m]	Re	Nu	Pressure Loss [Pa]
**1**	DSW2	0.03680	4627	326	544
**2**	DSW8	0.04657	5856	1072	5444
	**Double**	$D_{h}\;$ **[m]**	**Re**	**Nu**	**Pressure Loss [Pa]**
**1**	DDW28	0.02912	3662	487	2561
**2**	DDW38	0.02854	3589	440	1801
**3**	DDW48	0.02797	3517	372	1098
**4**	DDW58	0.02741	3446	357	914
**5**	DDW68	0.02680	3370	308	689

**Table 6:** Flow features and CFD results of gyroid structure.

	Single	$D_{h}$ [m]	Re	Nu	Pressure Loss [Pa]
**1**	GSW2	0.03956	4975	157	179
**2**	GSW8	0.04737	5956	461	1586
	**Double**	$D_{h}\;$ **[m]**	**Re**	**Nu**	**Pressure Loss [Pa]**
**1**	GDW28	0.03013	3789	322	1200
**2**	GDW38	0.02974	3740	327	778
**3**	GDW48	0.02938	3694	297	673
**4**	GDW58	0.02903	3650	301	556
**5**	GDW68	0.02867	3607	261	393

**Table 7:** Analysis conditions for the 12-inch chuck.

12-Inch Chuck	
**Flow Rate**	30 LPM
**Power**	3000 W

**Table 8:** CFD results for Heat 1 using diamond.

	Type (1)	Avg. Temp. [°C]		Type (2)	Avg. Temp. [°C]
**Heat 1**	DSW1	37.67	**Heat 1**	DSW2	36.82
**Heat 2**	DDW68	38.36	**Heat 2**	DDW68	38.04
**Heat 3**	DDW68	38.13	**Heat 3**	DDW68	38.04
**Heat 4**	DDW38	38.53	**Heat 4**	DDW38	38.38
**Heat 5**	DDW28	38.30	**Heat 5**	DDW28	38.27
**Heat 6**	DDW38	37.99	**Heat 6**	DDW38	37.95
**Temp. Difference**		**0.86**	**Temp. Difference**		**1.56**

**Table 9:** Design variable for each heat section.

	Design Variables
**Heat 1**	DSW1 , DSW2
**Heat 2**	DDW58 , DDW68
**Heat 3**	DDW58 , DDW68
**Heat 4**	DDW28 , DDW38
**Heat 5**	DDW28 , DDW38
**Heat 6**	DDW28 , DDW38

**Table 10:** Optimal design equation.

**Find**	**Sampling Point (*x*)**
**Minimize**	f(x)=max(Avg.Temp. of x)−min(Avg.Temp. of x)

**Table 11:** DOE table for diamond configured using DLHC.

	Heat 1	Heat 2	Heat 3	Heat 4	Heat 5	Heat 6	Response
**Variable Section**	Single 1 mm	Double 8-5, 8-6	Double 8-5, 8-6	Double 8-2, 8-3	Double 8-2, 8-3	Double 8-2, 8-3	-
**Point 1**	DSW1	DDW68	DDW68	DDW28	DDW28	DDW38	0.86
**Point 2**	DSW1	DDW58	DDW58	DDW38	DDW38	DDW38	0.71
**Point 3**	DSW1	DDW58	DDW58	DDW28	DDW28	DDW28	0.40
**Point 4**	DSW1	DDW58	DDW68	DDW28	DDW38	DDW38	0.53
**Point 5**	DSW1	DDW58	DDW58	DDW38	DDW28	DDW38	0.62
**Point 6**	DSW1	DDW68	DDW68	DDW38	DDW38	DDW28	0.32
**Point 7**	DSW1	DDW68	DDW58	DDW38	DDW38	DDW28	0.39

**Table 12:** Optimal design model for the diamond proposed by SAO.

	Heat 1	Heat 2	Heat 3	Heat 4	Heat 5	Heat 6	Response
**SAO 1**	DSW1	DDW58	DDW68	DDW38	DDW38	DDW28	**0.44**
**SAO 2**	DSW1	DDW68	DDW68	DDW28	DDW38	DDW28	**0.23**

**Table 13:** Boxplot data for SAO 2 applied to the diamond structure.

	Type	Ave. Temp	Median Temp	Difference (Ave. − Med)	IQR
**Heat 1**	DSW1	38.16	38.07	0.09	1.58
**Heat 2**	DDW68	38.36	38.19	0.17	1.33
**Heat 3**	DDW68	38.20	38.07	0.13	1.36
**Heat 4**	DDW28	38.38	38.30	0.08	1.15
**Heat 5**	DDW38	38.26	38.19	0.07	1.16
**Heat 6**	DDW28	38.20	38.11	0.08	1.15

**Table 14:** CFD results for Heat 1 using gyroid.

	Type (1)	Avg. Temp [°C]		Type (2)	Avg. Temp [°C]
**Heat 1**	GSW1	40.23	**Heat 1**	GSW2	39.78
**Heat 2**	GDW68	40.73	**Heat 2**	GDW68	40.81
**Heat 3**	GDW68	40.04	**Heat 3**	GDW68	40.13
**Heat 4**	GDW38	40.82	**Heat 4**	GDW38	41.09
**Heat 5**	GDW28	40.79	**Heat 5**	GDW28	41.05
**Heat 6**	GDW38	40.49	**Heat 6**	GDW38	41.76
**Temp. Difference**		0.78	**Temp. Difference**		1.98

**Table 15:** DOE table for gyroid configured using DLH.

	Heat 1	Heat 2	Heat 3	Heat 4	Heat 5	Heat 6	Response
**Variable Section**	Single 1 mm	Double 8-5, 8-6	Double 8-5, 8-6	Double 8-2, 8-3	Double 8-2, 8-3	Double 8-2, 8-3	-
**Point 1**	GSW1	GDW68	GDW68	GDW28	GDW28	GDW38	0.78
**Point 2**	GSW1	GDW58	GDW58	GDW38	GDW38	GDW38	1.31
**Point 3**	GSW1	GDW58	GDW58	GDW28	GDW28	GDW28	1.15
**Point 4**	GSW1	GDW58	GDW68	GDW28	GDW38	GDW38	1.04
**Point 5**	GSW1	GDW58	GDW58	GDW38	GDW28	GDW38	1.43
**Point 6**	GSW1	GDW68	GDW68	GDW38	GDW38	GDW28	0.66
**Point 7**	GSW1	GDW68	GDW58	GDW38	GDW38	GDW28	0.96

**Table 16:** Optimal design model for the gyroid proposed by SAO.

	Heat 1	Heat 2	Heat 3	Heat 4	Heat 5	Heat 6	Response
**SAO 1**	GSW1	GDW68	GDW68	GDW28	GDW38	GDW28	0.71
**SAO 2**	GSW1	GDW68	GDW68	GDW28	GDW28	GDW28	0.66

**Table 17:** Boxplot data for Point 6 applied to the gyroid.

	Type	Ave. Temp	Median Temp	Difference (Ave. − Med)	IQR
**Heat 1**	GSW1	40.23	39.90	0.33	1.97
**Heat 2**	GDW68	40.63	40.46	0.17	1.65
**Heat 3**	GDW68	40.17	40.00	0.17	1.59
**Heat 4**	GDW38	40.72	40.55	0.17	1.56
**Heat 5**	GDW38	40.82	40.64	0.18	1.49
**Heat 6**	GDW28	40.83	40.73	0.10	1.62

**Table 18:** Boxplot data for SAO 2 applied to the gyroid.

	Type	Ave. Temp	Median Temp	Difference (Ave. − Med)	IQR
**Heat 1**	GSW1	40.23	39.96	0.27	1.95
**Heat 2**	GDW68	40.61	40.46	0.15	1.67
**Heat 3**	GDW68	39.95	39.85	0.10	1.63
**Heat 4**	GDW28	40.48	40.31	0.17	1.52
**Heat 5**	GDW28	40.48	40.37	0.11	1.51
**Heat 6**	GDW28	40.54	40.29	0.25	1.58

## Data Availability

The original contributions presented in the study are included in the article; further inquiries can be to the corresponding author.
